# Construction and empirical testing of a verifiable food safety complaint risk-triage model using human foods complaint system data from the United States Food and Drug Administration

**DOI:** 10.3389/fnut.2026.1919174

**Published:** 2026-09-29

**Authors:** Jinming Chen

**Affiliations:** College of Food Science and Engineering, Shanxi Agricultural University, Taigu, Shanxi, China

**Keywords:** adverse events, complaint triage, food safety, machine learning, reproducibility of results, United States Food and Drug Administration

## Abstract

**Background:**

Food safety complaint systems contain large numbers of heterogeneous adverse-event reports, but surveillance use requires reproducible data sources and interpretable rules for ranking reports that may involve severe outcomes.

**Objective:**

To construct and test a transparent case-level logistic triage model that assigns FDA Human Foods Complaint System (HFCS) reports an urgent-priority score for earlier human review.

**Methods:**

The official HFCS quarterly workbook was downloaded from the FDA website on 2026-05-13, and case-based reports received from January 1, 2004 through December 31, 2025 were analyzed (*n* = 149,642). Predictors were derived from validated age and date fields, sex, suspected product descriptors, MedDRA preferred-term counts, symptom-domain indicators, report year, and event-to-receipt lag. Product-group proportions were summarized with unadjusted Wilson 95% confidence intervals and interpreted descriptively. A balanced logistic regression model was assessed on a stratified test set.

**Results:**

Urgent-priority outcomes occurred in 47,093 reports (31.47%). Death appeared in 17,005 reports and hospitalization in 23,405. The hospitalization proportion was 24.9% (95% CI, 24.5–25.3%) among dietary-supplement reports and 18.2% (16.3–20.3%) among infant/baby-food reports; these unadjusted proportions were not interpreted as population risk differences. The model achieved an AUC of 0.723, PR-AUC of 0.526, Brier score of 0.214, Brier skill score of 0.008, a calibration intercept of −0.03, and a calibration slope of 0.91 on 37,411 test cases. Symptom count and the infant/baby-food indicator had the largest positive model weights.

**Conclusion:**

HFCS data can support reproducible complaint triage. The model is suitable for ordering reports for human review, whereas product-group contrasts and raw predicted probabilities require cautious interpretation and temporal or external validation.

## 1 Introduction

Food safety surveillance requires methods that can identify complaint reports suggestive of severe harm while managing large volumes of heterogeneous submissions. The public health burden remains substantial: U.S. estimates indicate millions of foodborne illnesses, more than one hundred thousand hospitalizations, and thousands of deaths each year (1, 2). Global assessments similarly describe foodborne hazards as a major cause of morbidity and mortality (3, 4). These burdens support surveillance systems that can distinguish routine complaint traffic from submitted reports that may warrant rapid clinical, epidemiologic, or regulatory attention. Complaint data are valuable because they may capture adverse-event narratives before formal outbreak confirmation, but they are incomplete, variable in detail, and influenced by reporting behavior. Accordingly, complaint triage in this study refers to ranking submitted food-safety complaint reports for earlier human review, not estimating disease incidence in exposed populations or confirming that a food product caused the reported event.

Regulatory agencies and researchers increasingly use data integration, predictive analytics, and machine-learning tools to support risk-based food safety oversight. FDA’s New Era of Smarter Food Safety emphasizes traceability, predictive analytics, faster outbreak response, and modern data tools (5). Technical guidance and surveillance reviews indicate that early-warning workflows can draw on structured surveillance data, inspection records, adverse-event reports, supply-chain information, and public data streams (6–8). Machine-learning studies further support risk-based prioritization in dynamic food-chain monitoring and regulatory policy analysis (9–11). Recent reviews extend these applications to food-safety monitoring, prediction, and AI-assisted decision support (12–14). Risk-notification databases can also be used for principle-based governance and AI-driven food-safety risk assessment (15, 16). The model evaluated here is a transparent case-level logistic risk-triage model: it uses variables available in a complaint record to estimate the probability that the report contains an urgent-priority outcome. The resulting score is intended to order complaints within a review queue, so that records with stronger severe-outcome patterns can be examined earlier by analysts.

The FDA Human Foods Complaint System (HFCS) provides a public source for studying complaint triage in products regulated by the FDA Human Foods Program. The HFCS quarterly workbook includes case-based and product-based views with demographic, product, symptom, and outcome fields for reports involving foods, dietary supplements, infant formula, and related products (17, 18). Symptoms are coded using Medical Dictionary for Regulatory Activities (MedDRA) preferred terms, which supports consistent retrieval and analysis of reported adverse-event concepts (19). The case-based structure is suitable for modeling because each row can be treated as a single review unit with one set of reported patient, product, symptom, timing, and outcome attributes.

HFCS reports enter the system through voluntary reports from consumers and health care practitioners, mandatory serious-adverse-event reports from dietary supplement firms, and other FDA reporting channels (17, 20, 21). These data provide a structured setting for evaluating whether routinely available complaint fields can help rank cases for urgent review. Within this surveillance setting, the relevant unit for prioritization is the individual case report rather than an outbreak, a product lot, or an exposed population. A transparent model is especially appropriate because analysts need to understand which reported characteristics contribute to a priority score before that score affects review workflow.

This study constructed and internally tested a case-level food safety complaint risk-triage model using the FDA HFCS case-based data file. The model was designed to assign each report an urgent-priority probability for review ordering, not to determine causality or estimate incidence. The aims were to describe temporal and product-group patterns in HFCS reports, define an urgent-priority outcome from FDA outcome terms, engineer triage predictors from demographic, product, symptom, and timing fields, and evaluate whether a balanced logistic regression model can rank reports for urgent-priority review. The study addresses four analytic questions: how HFCS reports vary over time, how unadjusted urgent-priority proportions vary across operational product groups, which reported features receive the largest model weights, and how accurately a transparent model can prioritize cases for review.

## 2 Materials and methods

### 2.1 Study design and data source

#### 2.1.1 Verifiable retrospective design

This retrospective observational study analyzed case-level FDA HFCS adverse-event reports. Each analytic record represented one HFCS case from the case-based worksheet. No individual contact, intervention, or product testing was performed. The design was intended to assess a surveillance triage framework for reported complaints, not to adjudicate product causation or evaluate the clinical management of individual events. The unit of inference was therefore the submitted report, and all findings are interpreted as patterns among reported HFCS cases rather than as population rates among exposed consumers.

#### 2.1.2 Source retrieval and extraction time

The FDA HFCS Quarterly Data Excel workbook was downloaded from the FDA HFCS data page on 2026-05-13. The FDA documentation describes the file as a public Excel extract containing demographic, administrative, product, symptom, and patient outcome information (17, 18). The accompanying readme stated that the extract covered reports received by HFCS from January 1, 2004 through December 31, 2025. The downloaded workbook was retained as the analysis source file, and all tabulations and model outputs were generated from its case-based worksheet.

#### 2.1.3 Inclusion and exclusion criteria

Rows from the case-based worksheet were eligible when the FDA first-received date was from 2004-01-01 to 2025-12-31. Records outside the documented coverage period were not included in analysis. Records with missing age, sex, event date, product text, or symptom information were retained because incomplete fields are expected in complaint and adverse-event reporting systems. Missing categorical values were coded as explicit unknown categories when the field was used directly. Continuous fields followed deterministic validity rules detailed in Supplementary methods: age was numerically coerced, converted to years from reported day, week, month, or year units, and set to missing when negative, greater than 120 years, or paired with an unrecognized unit; date fields were parsed with invalid values coerced to missing; event-to-receipt lag was set to missing when negative and was capped at 3,650 days. Continuous missing values were imputed within the training pipeline. After date filtering, the analytic dataset contained 149,642 case-level reports.

### 2.2 Variable construction

#### 2.2.1 Outcome definition

The primary outcome was an urgent-priority flag. A case was coded 1 if the patient outcome text included death, life-threatening outcome, hospitalization, disability, congenital anomaly, or required intervention, and 0 otherwise. These components were selected before modeling because they represent severe reported outcomes that would normally justify earlier surveillance review (20, 21). Death, hospitalization, life-threatening outcome, and disability were also summarized separately so that the composite outcome could be interpreted through its main components.

#### 2.2.2 Predictor construction

Predictors were limited to fields available at initial complaint triage. Age was expressed in years, and sex was normalized to female, male, or unknown. Suspect-product strings were parsed from left to right for bracketed FDA Product_Code-Description blocks, and the first recognized suspect-product industry description was mapped to eight operational groups: dietary supplements, infant/baby foods, beverages, produce/seafood/nuts, bakery/snacks/confectionery, dairy/egg/meat, food additives, and other foods/products. The produce/seafood/nuts stratum was pooled to control dimensionality and stabilize estimation; it does not imply that these products share biological hazards or harm profiles. Other foods/products was a residual category that could include prepared foods and descriptors not captured by the seven named groups. The dairy/egg/meat label refers only to products represented in an FDA-regulated dataset; most meat and poultry from amenable species fall primarily under U.S. Department of Agriculture Food Safety and Inspection Service jurisdiction and are not represented as a general meat-market denominator (22).

#### 2.2.3 Symptom and timing features

MedDRA preferred-term strings were split at commas, trimmed, and counted after empty tokens were removed. Case-insensitive regular-expression indicators identified allergy or hypersensitivity, gastrointestinal, neurological, respiratory, and cardiac symptom domains. Suspect and concomitant-product counts were calculated from nonempty product entries in the corresponding strings. Event-to-receipt lag was the number of days between event date and FDA first-received date when both dates were valid; negative values were treated as missing and nonnegative values were capped at 3,650 days. Report year and month were derived from FDA first-received date. These deterministic rules, including unit conversion, tokenization, and missing-value handling, are specified in Supplementary methods so that the variables can be reproduced from the public workbook.

### 2.3 Statistical analysis and modeling

#### 2.3.1 Descriptive analysis

Descriptive statistics covered record counts, urgent-priority proportion, component outcomes, product group, age, symptom count, and reporting lag. Skewed continuous variables were summarized by medians. Product-group tables reported counts, urgent-priority proportion, death proportion, hospitalization proportion, median age, and median symptom count. Unadjusted Wilson 95% confidence intervals were calculated for key product-group proportions to quantify sampling precision within the reported-case dataset. No pairwise hypothesis tests or adjusted product-group risk comparisons were used; product-group statements were therefore restricted to descriptive observations and were not interpreted as evidence of population-level risk differences. Violin plots, boxplots, and heat maps displayed distributional spread, overlap between outcome groups, and temporal concentration of reports. Percentages were calculated using the filtered analytic dataset as the denominator unless otherwise specified.

#### 2.3.2 Model specification and validation

A balanced logistic regression model was fitted to estimate the probability that a complaint report met the urgent-priority definition. Inputs included age, sex, product group, suspected and concomitant product counts, symptom count, symptom-domain indicators, capped event-to-receipt lag, and report year. Categorical features were one-hot encoded, and continuous features were median-imputed and standardized within the training pipeline. A stratified 75/25 train-test split with random seed 2,026,051 was used. Test-set performance was assessed by AUC, PR-AUC, accuracy, sensitivity, precision, F1 score, Brier score, Brier skill score relative to a prevalence-only prediction, and the 0.50-threshold confusion matrix (23–25). Calibration was evaluated on the held-out test set using a ten-bin plot of observed urgent-priority proportions against mean predicted probabilities. Calibration intercept and slope were estimated from a logistic recalibration model using the logit of the predicted probability, with the 45-degree line representing ideal calibration. Balanced class weights were used because urgent-priority outcomes were less frequent than non-urgent outcomes. Coefficients were treated as regularized predictive weights rather than inferential effect estimates; exponentiated weights were provided to aid interpretation, but confidence intervals and causal odds-ratio claims were not assigned to them.

#### 2.3.3 Reproducibility information and supplementary materials

The source FDA HFCS workbook is publicly available from the FDA sources cited in references (17, 18) and is not redistributed with this submission. The materials supplied with the manuscript comprise one Supplementary document titled Supplementary materials, containing Supplementary methods and Supplementary Table S1, together with Supplementary Figure S1 and Supplementary Figure S2. Supplementary methods document the age-unit conversions, date validation, lag capping, product parsing, symptom tokenization, domain rules, and training-fold imputation. Supplementary Table S1 reports coefficients and exponentiated model weights, Supplementary Figure S1 presents held-out test-set calibration, and Supplementary Figure S2 presents the 0.50-threshold confusion matrix (17, 18, 24). No additional processed datasets, analysis scripts, CSV output tables, codebook, readme, or analysis log are included.

## 3 Results

### 3.1 Data source structure and temporal coverage

The official workbook contained 149,945 rows in the case-based worksheet. After restricting the FDA first-received date to the documented extract period, 149,642 cases remained for analysis. The earliest included FDA first-received date was 2004-01-02, and the latest was 2025-12-31. Table 1 presents the annual distribution of records and urgent-priority component counts. The number of reports increased over time, with particularly high volumes in 2016, 2017, and 2021. The annual urgent-priority rate varied substantially, supporting temporal monitoring rather than a single static prevalence estimate. Because HFCS is a reporting system rather than an exposure registry, the annual counts should be read as reporting volume and case mix, not as incidence in the population.

**Table 1:** Complete annual summary of HFCS case-level reports used in the analysis.

Year	Records	Urgent cases	Urgent rate	Death cases	Hospitalization cases
2004	2,738	575	0.210	53	332
2005	2027	400	0.197	34	251
2006	1996	301	0.151	21	185
2007	3,077	490	0.159	28	298
2008	2,947	716	0.243	19	451
2009	4,368	968	0.222	40	688
2010	3,822	832	0.218	33	589
2011	4,776	896	0.188	63	650
2012	5,519	1,337	0.242	101	869
2013	6,530	1,657	0.254	132	1,121
2014	5,402	1,377	0.255	69	930
2015	7,607	2064	0.271	279	1,239
2016	10,204	2,627	0.257	590	1,482
2017	12,906	3,451	0.267	1838	1,187
2018	9,173	3,248	0.354	1736	1,190
2019	8,279	2,755	0.333	1,331	1,023
2020	9,368	3,705	0.395	2,162	1,140
2021	17,089	8,475	0.496	5,008	3,611
2022	11,466	4,145	0.362	1,151	2,464
2023	8,813	3,822	0.434	2065	1,456
2024	5,265	1757	0.334	120	1,143
2025	6,270	1,495	0.238	132	1,106

Figure 1 shows the distribution of patient age across suspected product groups. Dietary supplements and the residual other-foods/products group had older median age profiles in the submitted reports, whereas infant/baby foods were concentrated near infancy by construction. The broad within-group dispersion supports retaining age as a continuous predictor. These distributions describe the HFCS case mix and do not compare age-specific population risks across products.

**Figure 1:**
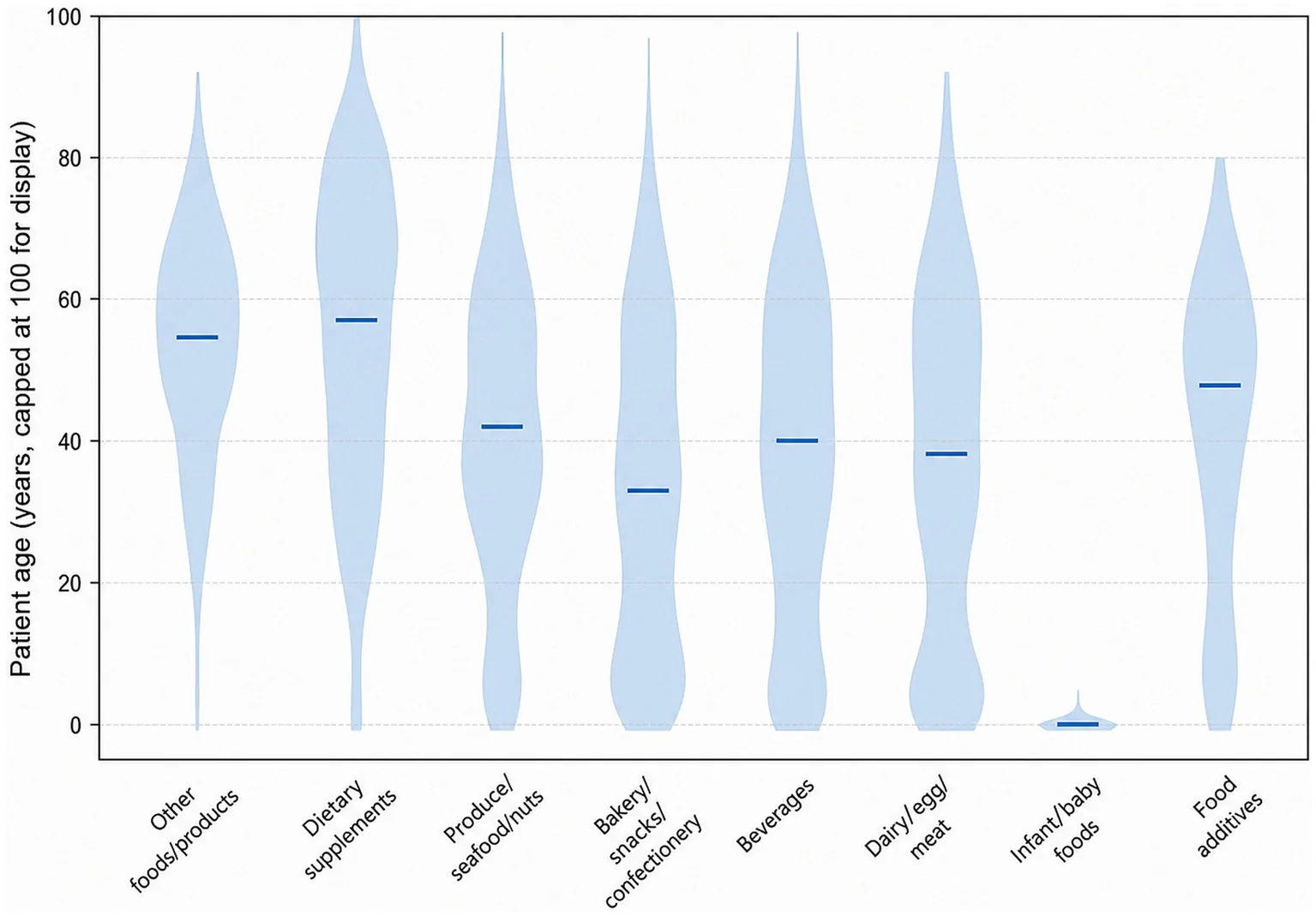
Violin plot of age distribution by suspected product group. Medians are shown inside each violin; age is capped at 100 years for display only.

### 3.2 Product-group heterogeneity

Table 2 summarizes unadjusted product-group distributions. Other foods/products and dietary supplements were the largest groups. The urgent-priority proportions were 38.5% (95% CI, 38.1–38.9%) for other foods/products, 35.4% (35.0–35.8%) for dietary supplements, 32.3% (28.3–36.6%) for food additives, and 27.0% (24.8–29.4%) for infant/baby foods. Hospitalization proportions were 24.9% (24.5–25.3%) for dietary supplements and 18.2% (16.3–20.3%) for infant/baby foods, while the death proportion in the broad other-foods/products group was 25.7% (25.4–26.1%). These confidence intervals quantify precision of unadjusted reported-case proportions; they were not used as pairwise significance tests and do not establish product-specific incidence or causal risk. The residual group includes heterogeneous and potentially prepared-food descriptors, and the produce/seafood/nuts category is an operational consolidation rather than a common-hazard class.

**Table 2:** Product-group summary of HFCS reports with unadjusted Wilson 95% confidence intervals.

Product group	Records	Urgent % (95% CI)	Death % (95% CI)	Hosp. % (95% CI)	Median age	Median symptom count
Other foods/products	59,866	38.5 (38.1–38.9)	25.7 (25.4–26.1)	12.8 (12.5–13.1)	53	2
Dietary supplements	50,919	35.4 (35.0–35.8)	2.7 (2.6–2.8)	24.9 (24.5–25.3)	57	2
Produce/seafood/nuts	17,655	14.3 (13.8–14.8)	0.7 (0.6–0.8)	7.8 (7.4–8.2)	43	3
Bakery/snacks/confectionery	10,433	15.9 (15.2–16.6)	0.3 (0.2–0.4)	6.9 (6.4–7.4)	33	2
Beverages	5,049	15.1 (14.1–16.1)	0.5 (0.3–0.7)	8.2 (7.5–9.0)	40	2
Dairy/egg/meat	3,780	14.0 (12.9–15.1)	0.3 (0.2–0.5)	5.7 (5.0–6.5)	38	3
Infant/baby foods	1,454	27.0 (24.8–29.4)	2.6 (1.9–3.6)	18.2 (16.3–20.3)	0.58	2
Food additives	486	32.3 (28.3–36.6)	1.6 (0.8–3.2)	11.3 (8.8–14.4)	44	3

Rate columns show percentages with unadjusted Wilson 95% CIs; intervals describe precision within reported cases and are not pairwise tests.

Figure 2 shows symptom-count distributions by product group and urgent-priority status. Urgent-priority cases generally had equal or higher symptom-count distributions than non-urgent cases, although overlap was substantial in every product group. In the urgent-priority other-foods/products stratum, the first quartile, median, and third quartile were all two preferred terms, so the interquartile box collapsed to a line; the figure uses a narrow visible box at that value and the legend states this explicitly. The extensive overlap indicates that triage cannot rely on symptom count alone and must combine symptom burden with age, product group, symptom domain, and timing information.

**Figure 2:**
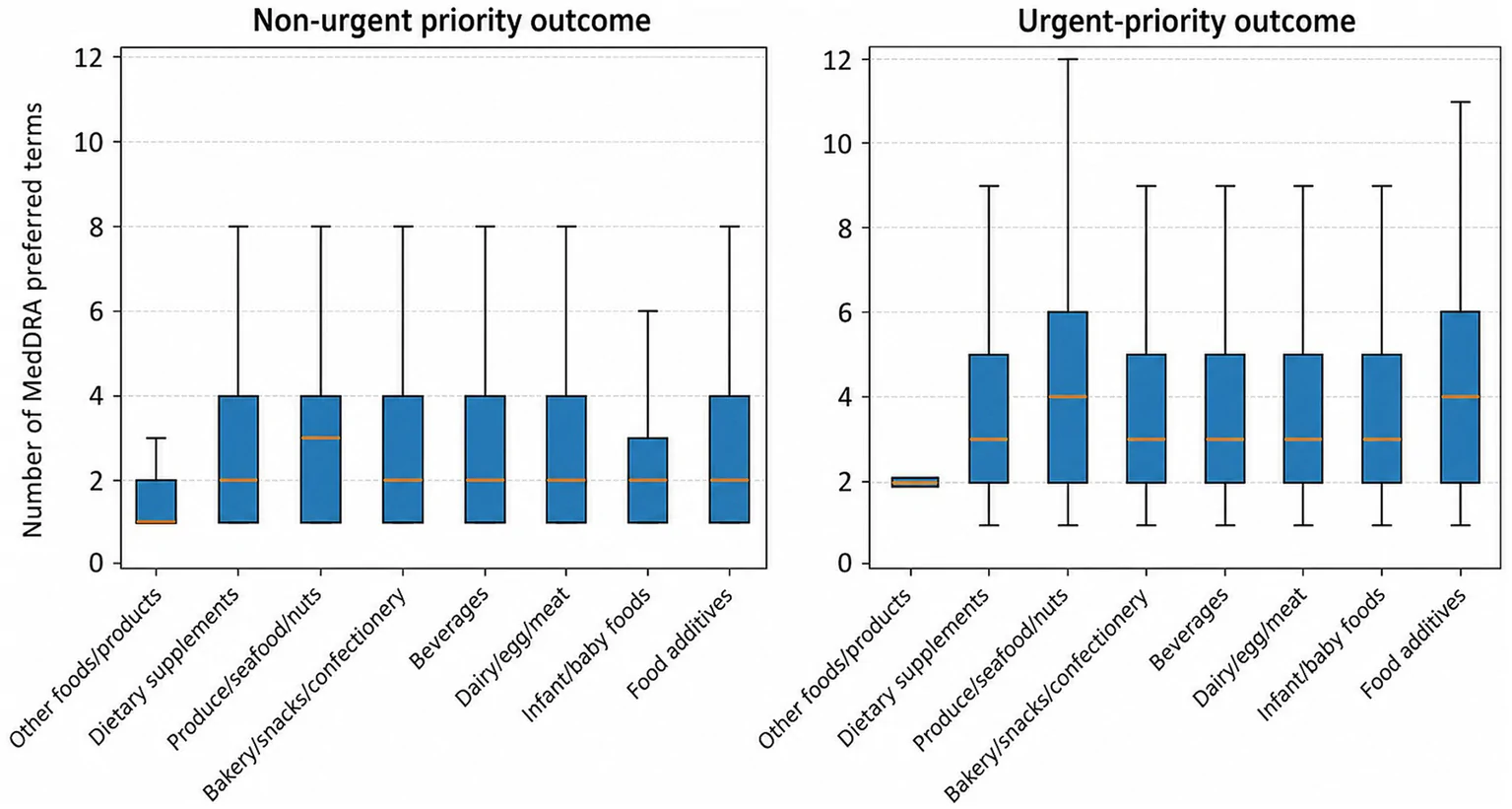
Boxplots of MedDRA preferred-term counts by product group and urgent-priority status. Extreme values are suppressed for readability. For urgent-priority other foods/products, q1, median, and q3 were all 2, so the box collapses at 2; a narrow box is displayed only to make the zero interquartile range visible.

### 3.3 Outcome composition and temporal density

Table 3 presents the outcome components used to construct the urgent-priority flag. Urgent-priority outcomes were present in 47,093 reports, representing 31.47% of the analytic sample. Hospitalization appeared in 23,405 reports and death appeared in 17,005 reports. Component counts overlap because a single case may list multiple outcomes.

**Table 3:** Outcome components used in the urgent-priority definition.

Outcome component	Cases	Percent of cases
Death	17,005	11.36
Hospitalization	23,405	15.64
Life threatening	6,041	4.04
Disability	4,734	3.16
Any urgent-priority outcome	47,093	31.47

Percentages do not sum to the composite percentage because outcome components can overlap within a case.

Figure 3 displays the monthly density of case reports by year. The heat map reveals strong temporal variation, including a visible rise in the later years of the dataset. This variation likely reflects changes in reporting behavior, product mix, system operations, and public awareness in addition to any true changes in adverse-event occurrence. Therefore, report volume was used descriptively rather than interpreted as incidence. The visual pattern also supports including report year in the triage model, because the meaning of a complaint record may depend partly on the reporting environment in which it was submitted.

**Figure 3:**
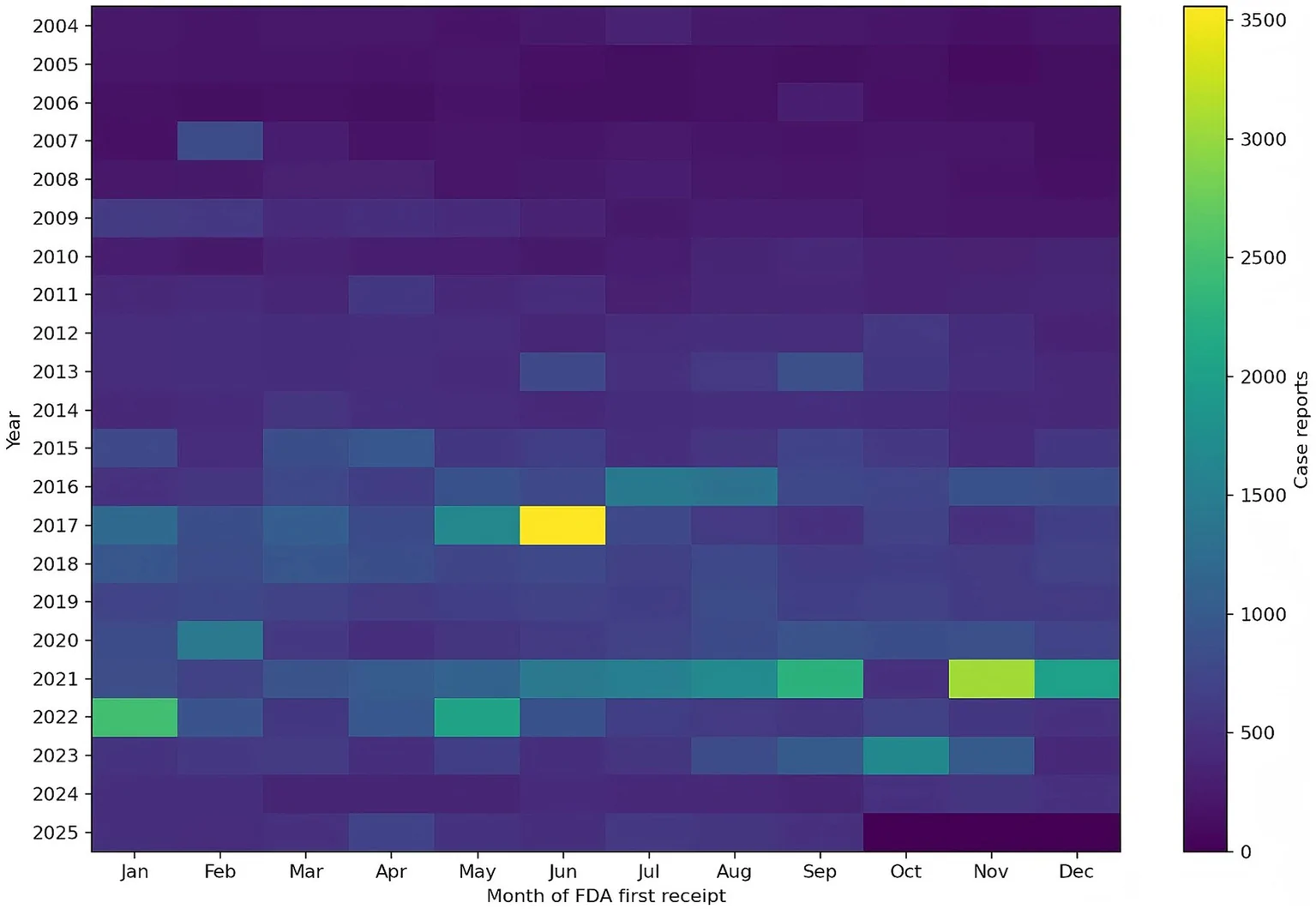
Heat map of monthly case-report density by FDA first-received year and month.

Figure 4 presents the monthly urgent-priority rate. Compared with the volume heat map, the rate heat map shows that high reporting volume and high urgent-priority proportion do not always coincide. This distinction is important for surveillance triage because a large number of reports may reflect reporting intensity, while the urgent-priority rate reflects outcome severity within the reported cases.

**Figure 4:**
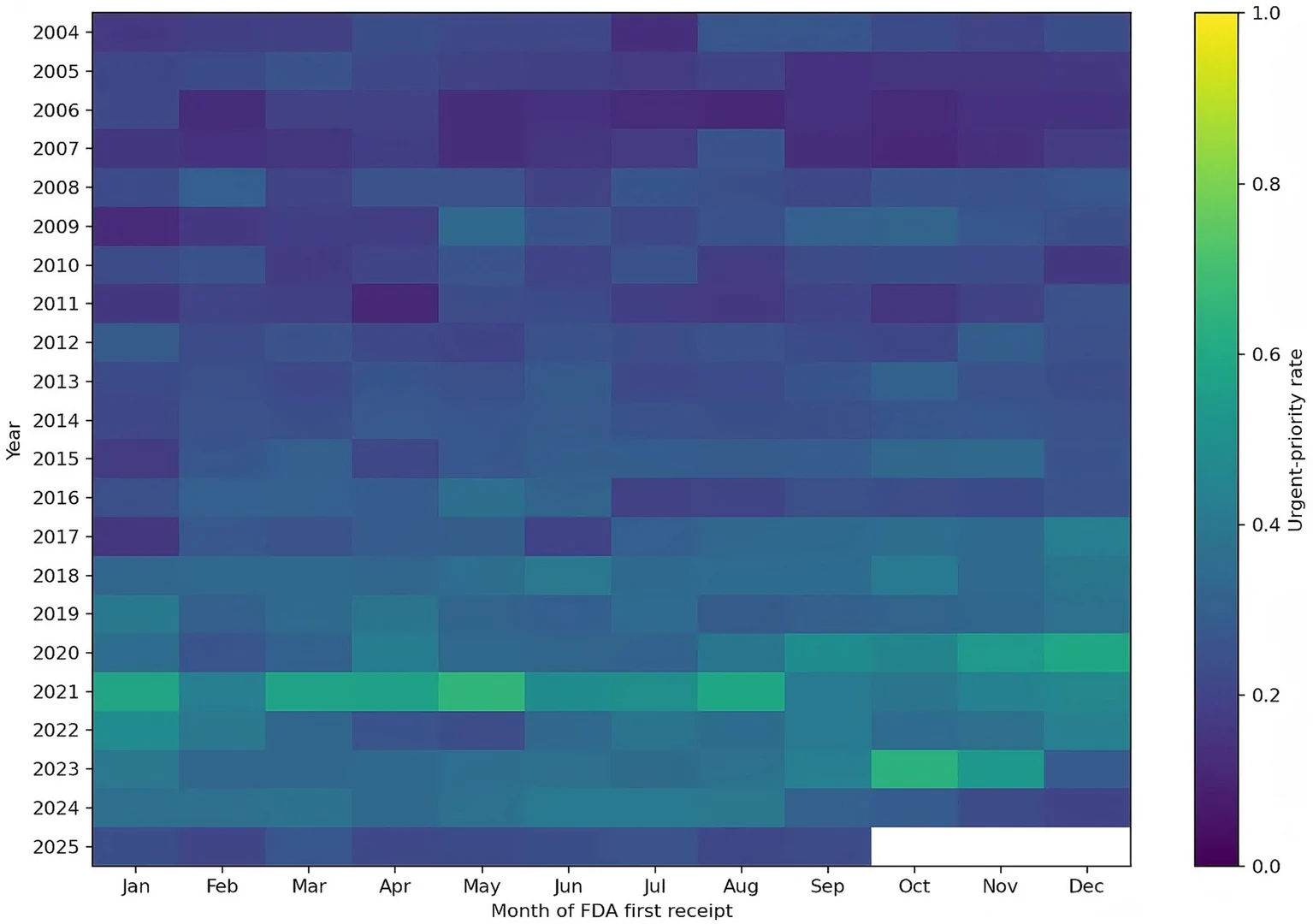
Heat map of monthly urgent-priority outcome rate by FDA first-received year and month.

### 3.4 Risk-triage model performance and interpretation

Table 4 reports model performance on the stratified test set. The balanced logistic model achieved an AUC of 0.723 and a PR-AUC of 0.526. At the 0.50 probability threshold, sensitivity was 0.703 and precision was 0.477. The Brier score was 0.214, compared with 0.216 for a prevalence-only prediction based on the test prevalence of 0.315, yielding a Brier skill score of 0.008. The held-out calibration intercept was −0.03 and the calibration slope was 0.91. The near-zero intercept indicated little overall calibration offset, whereas the slope below 1 indicated mildly over extreme predictions. The binned calibration curve followed the ideal line through much of the probability range, with more visible over prediction in the highest-probability bins (Supplementary Figure S1). Thus, the model improved rank discrimination more clearly than overall probability accuracy, and its raw probabilities should still be interpreted cautiously outside this internal test set.

**Table 4:** Test-set performance of the balanced logistic urgent-priority triage model.

AUC	PR-AUC	Accuracy	Sensitivity	Precision	F1	Brier score	Test n	Test prevalence
0.723	0.526	0.664	0.703	0.477	0.569	0.214	37,411	0.315

The largest positive model weights were infant/baby foods [coefficient 0.702; exp.(coefficient) 2.02], standardized symptom count (0.546; 1.73), other foods/products (0.401; 1.49), dietary supplements (0.375; 1.46), standardized report year (0.287; 1.33), and standardized capped event-to-receipt lag (0.262; 1.30). The largest negative weights were beverages (−0.603; 0.55), produce/seafood/nuts (−0.536; 0.59), and dairy/egg/meat (−0.528; 0.59) (Supplementary Table S1). Operationally, higher symptom burden and selected product indicators moved reports upward in the review queue, whereas negative weights moved otherwise similar reports downward. Continuous-predictor transformations were standardized; one-hot category weights are regularized model parameters, not pairwise adjusted odds ratios or causal product effects. The 0.50-threshold confusion matrix showed higher sensitivity than precision (Supplementary Figure S2).

## 4 Discussion

FDA HFCS data supported an empirical workflow in which routinely available complaint fields were converted into a review-priority score for individual reports. The analysis linked case-level records, deterministic demographic and symptom features, operational product-group summaries, temporal displays, and an interpretable logistic prediction model within one official data source. The resulting outputs characterize severe-outcome reporting patterns and provide an auditable basis for queue ordering. Their scientific value lies in transparent surveillance prioritization rather than estimation of product-specific disease burden.

The unadjusted product-group summaries showed heterogeneous reported-case proportions, but the contrasts require restrained interpretation. Dietary-supplement reports had a relatively large hospitalization proportion and infant/baby-food reports had a relatively high urgent-priority proportion within HFCS, with confidence intervals reported to show precision rather than to establish pairwise risk differences. Reporting mandates, consumer behavior, product use, and case severity can all affect these proportions. The produce/seafood/nuts group was created for operational stability and should not be read as a shared-hazard category; seafood should be separated in future expert-coded mappings. The residual other-foods/products group is particularly broad and may conceal prepared-food or other subcategory signals. Likewise, dairy/egg/meat represents only FDA-jurisdiction products in this dataset and not the majority of meat and poultry overseen by USDA-FSIS (22).

The model achieved moderate discrimination in a noisy passive-surveillance setting. The AUC of 0.723 indicates useful ranking beyond chance, while the PR-AUC of 0.526 is informative under class imbalance (23). Probability performance was less compelling: the Brier skill score of 0.008 indicated only a small improvement over the prevalence-only reference. Internal test-set calibration was nevertheless close to ideal overall, with an intercept of −0.03 and a slope of 0.91. The slope below 1 and the deviation in the highest-probability bins indicate mild over extremity, so model output is best used as a relative queue score. Calibration should be re-evaluated in temporally separated or external data, and recalibration should be performed if reporting practices or case mix change.

The results are consistent with the broader literature on data-driven food safety surveillance. Machine learning can support risk-based prioritization when it is used carefully and paired with transparent governance (5–7). Big-data, inspection, and supply-chain studies further support dynamic food-chain risk management and regulatory policy analysis (8, 9). Online and complaint-based data can reveal signals that conventional inspection systems may not capture immediately, but they require strong caveats about bias, representativeness, and false alarms (10, 11). Recent reviews have extended these applications to food-safety monitoring, prediction, and AI-assisted decision support (12–14). Governance and RASFF-based studies emphasize principle-based oversight and AI-driven food-safety risk assessment (15, 16). The present analysis therefore treats HFCS as a surveillance signal repository, not as a causal epidemiologic cohort. This distinction is essential because the FDA explicitly cautions that HFCS reports are as submitted, may contain incomplete information, cannot prove that a product caused an event, and cannot be used to estimate incidence or risk (17, 18).

A principal strength is the transparency of the data source, variable definitions, and modeling pipeline. Documented HFCS fields remain linked to deterministic transformations, descriptive summaries, model inputs, and output diagnostics. This approach is consistent with prediction-model reporting guidance emphasizing explicit data sources, predictor definitions, outcomes, modeling procedures, discrimination, calibration, and validation (25). Interpretability is also operationally important: coefficient direction and magnitude, symptom-count distributions, product-group summaries, and heat maps can be audited by analysts and supervisors more readily than opaque decision rules.

Several limitations constrain interpretation and deployment. HFCS is a passive and mixed reporting system in which reporting probability varies by product, severity, reporting mandate, media attention, and awareness. Product groups are rule-based operational strata rather than biologically homogeneous exposure categories, and no adjusted or multiplicity-controlled product-group comparisons were performed. The urgent-priority outcome is derived from reported outcome text and was not independently adjudicated. Random train-test splitting provides internal validation but does not test distribution shift across reporting eras; temporal validation that trains on earlier years and tests on later years is the next essential evaluation because report year itself carried model weight. Calibration intercept, slope, and a binned calibration plot were assessed only in the internal held-out test set; temporal or external calibration and any required recalibration remain unavailable. Logistic regression was selected for interpretability and was not benchmarked against nonlinear methods; future studies should compare penalized logistic regression, random forests, and gradient boosting under the same temporally separated validation and calibration framework. Finally, the model lacks exposure denominators, laboratory confirmation, robust duplicate detection, and detailed clinical covariates, and comparisons with archived FDA recall or enforcement data remain future work (25, 26).

For operational evaluation, high-scoring reports could enter an earlier analyst-review queue while routine reports remain available for standard review. Implementation should be prospective and governed by predeclared workload thresholds, subgroup performance checks, temporal calibration monitoring, duplicate detection, product-identity verification, laboratory evidence, outbreak investigation, and recall information. A multi-source benchmark could add archived openFDA enforcement data, recall communications, local complaints, and public-attention indicators, while comparing interpretable and nonlinear models under identical temporal splits (26). Human review remains the decision point because the present score summarizes report patterns rather than regulatory evidence.

## 5 Conclusion

This study constructed and internally tested a verifiable food safety complaint risk-triage model using 149,642 FDA HFCS case-level reports received from 2004-01-02 through 2025-12-31. The model achieved an AUC of 0.723 and PR-AUC of 0.526, while a Brier skill score of 0.008 showed limited improvement in overall probability accuracy over a prevalence-only reference. The internal calibration intercept of −0.03 and slope of 0.91 indicated limited overall offset with mild over extremity. Product-group proportions and coefficient magnitudes are informative for describing case mix and queue behavior but do not establish causal or population-risk differences. The framework is therefore most appropriate for auditable review ordering, with temporal or external validation, prospective calibration monitoring, refined product coding, and comparison with alternative algorithms required before operational deployment.

## Data Availability

The original contributions presented in the study are included in the article/Supplementary material, further inquiries can be directed to the corresponding author.

## Supplementary material

SUPPLEMENTARY FIGURE S1 Calibration of predicted urgent-priority probability in the held-out test set. Points show observed urgent-priority rates plotted against mean predicted probabilities in ten probability bins; vertical error bars show uncertainty intervals for observed bin rates. The dashed 45-degree line indicates ideal calibration. Test prevalence was 0.315, the Brier score was 0.214, the prevalence-reference Brier score was 0.216, the Brier skill score was 0.008, the calibration intercept was −0.03, and the calibration slope was 0.91.

SUPPLEMENTARY FIGURE S2 Confusion matrix for the balanced logistic urgent-priority model at the 0.50 probability threshold in the stratified test set.
