# Supplementary material for: Construction and empirical testing of a verifiable food safety complaint risk-triage model using human foods complaint system data from the United States Food and Drug Administration
Source: Front Nutr. 2026 Sep 29;13:1919174. doi: 10.3389/fnut.2026.1919174 (PMC13657942; doi:10.3389/fnut.2026.1919174)
Supplement: Supplementary file 1 [file Supplementary_File_1.DOCX]

Supplementary Materials

**Supplementary Methods: Parsing and validation rules**

The Date_FDA_First_Received_Case and Date_Event fields were parsed as calendar dates, with unparseable entries coerced to missing. Patient_Age was coerced to numeric after surrounding whitespace was removed. Age_Units was normalized case-insensitively, and age was converted to years using day/365.25, week/52.1775, month/12, and year unchanged. Missing or unrecognized units, negative converted ages, and values greater than 120 years were assigned missing age; no report was excluded solely because age was unavailable.

Sex labels were normalized case-insensitively to Female, Male, or Unknown. Blank, ambiguous, and unrecognized values were assigned Unknown.

Suspect_Products and Concomitant_Products were treated as raw strings. Product counts were the number of nonempty product entries identified by the workbook delimiters; a nonempty string without a delimiter counted as one. Bracketed Product_Code-Description blocks were parsed from left to right, and the first recognized suspect-product description was mapped to the eight operational groups. If no recognized descriptor was present, the report was assigned to Other foods/products. Produce/seafood/nuts was pooled for dimensionality control and model stability rather than biological similarity. Dairy/egg/meat reflects products represented under FDA jurisdiction and does not represent most amenable meat and poultry products regulated by USDA-FSIS.

Case_MedDRA_Preferred_Terms was split at commas. Leading and trailing whitespace and empty tokens were removed, and the remaining terms were counted. Case-insensitive regular expressions assigned allergy/hypersensitivity, gastrointestinal, neurological, respiratory, and cardiac indicators using corresponding preferred-term language such as anaphylaxis or urticaria; abdominal, nausea, vomiting, or diarrhea terms; headache, dizziness, seizure, or syncope terms; dyspnea, breathing, wheeze, or cough terms; and chest-pain, palpitation, or tachycardia terms.

Event-to-receipt lag was Date_FDA_First_Received_Case minus Date_Event in calendar days. A missing or invalid date produced a missing lag, negative lags were assigned missing, and nonnegative values were capped at 3,650 days. Report year and month were derived from Date_FDA_First_Received_Case. Continuous missing values were median-imputed using the training partition only and were standardized using training-partition parameters before application to the test partition.

**Coefficient interpretation**

Continuous predictors were standardized, so exp(coefficient) for a continuous feature is the fitted odds multiplier for a one-standard-deviation increase, conditional on the other encoded predictors. For one-hot categorical indicators, exponentiated values are transformations of regularized predictive weights and are not pairwise adjusted product-risk odds ratios against a single causal reference category. Coefficients were reported for model interpretation rather than null-hypothesis inference.

**Supplementary Table S1. Top model coefficients.**

| feature | coefficient | exp(coefficient) |
| --- | --- | --- |
| cat__product_group_Infant/baby foods | 0.702 | 2.018 |
| cat__product_group_Beverages | -0.603 | 0.547 |
| num__symptom_count | 0.546 | 1.726 |
| cat__product_group_Produce/seafood/nuts | -0.536 | 0.585 |
| cat__product_group_Dairy/egg/meat | -0.528 | 0.590 |
| cat__product_group_Other foods/products | 0.401 | 1.493 |
| cat__SEX_Unknown | -0.391 | 0.676 |
| cat__product_group_Dietary supplements | 0.375 | 1.455 |
| cat__product_group_Bakery/snacks/confectionery | -0.341 | 0.711 |
| cat__product_group_Food additives | 0.289 | 1.335 |
| num__report_year | 0.287 | 1.332 |
| num__event_lag_days_capped | 0.262 | 1.300 |
| num__symptom_gastro | -0.248 | 0.780 |
| cat__SEX_Male | 0.211 | 1.235 |
| num__symptom_respiratory | -0.156 | 0.856 |
| num__symptom_cardiac | 0.118 | 1.125 |
| num__age_years | 0.079 | 1.082 |
| num__symptom_neuro | -0.074 | 0.929 |
| num__suspect_product_count | 0.071 | 1.074 |
| num__symptom_allergy | -0.063 | 0.939 |

Note: Coefficients are regularized predictive weights. Continuous predictors were standardized; categorical predictors were one-hot encoded. Exponentiated values are provided for scale interpretation and are not causal effect estimates.
